# Supplementary material for: Protocol for the REVELIO test-track pilot study: A randomised, controlled, single-centre trial in healthy recreational cannabis users investigating real-time in-vehicle detection of cannabis-impaired driving
Source: PLoS One. 2026 Oct 1;21(10):e0349328. doi: 10.1371/journal.pone.0349328 (PMC13630308; doi:10.1371/journal.pone.0349328)
Supplement: Supplementary file 3 [file pone.0349328.s003.pdf]

Dear PLOS ONE Editorial Office,

Thank you very much for your feedback on our manuscript. We appreciate your guidance, which helped us strengthen the manuscript and align it more closely with the journal's submission requirements. Following the editorial review, we have addressed the requested points as follows:

i) The manuscript is based on study protocol version 5.1, which is the current amended study protocol followed during study execution and was included in the initial submission. In response to your request, we have additionally uploaded protocol version 4.0, which was reviewed at the initial ethics submission. We have also uploaded the ethics committee correspondence confirming receipt/acknowledgement of protocol version 5.1.

ii) We have revised the "Data collection" subsection to include additional methodological detail directly in the manuscript, including further clarification of the study procedures and data collection processes across study visits.

iii) We have revised the "Statistical analysis" section to provide additional methodological detail on the planned machine-learning analyses, including further clarification of the feature engineering and selection approach, as well as the planned model validation and performance evaluation procedures.

Thank you again for your consideration. We look forward to your further feedback.

Sincerely,
Michal Bechny
on behalf of all authors
